# Epigallocatechin Gallate (EGCG) Is the Most Effective Cancer Chemopreventive Polyphenol in Green Tea

**DOI:** 10.3390/nu4111679

**Published:** 2012-11-08

**Authors:** Guang-Jian Du, Zhiyu Zhang, Xiao-Dong Wen, Chunhao Yu, Tyler Calway, Chun-Su Yuan, Chong-Zhi Wang

**Affiliations:** 1 Tang Center for Herbal Medicine Research, University of Chicago, Chicago, IL 60637, USA; Email: gdu@dacc.uchicago.edu (G.-J.D.); zhiyu@uchicago.edu (Z.Z.); cpuwxd@gmail.com (X.-D.W); chunhaoyu@hotmail.com (C.Y.); tyler.calway@gmail.com (T.C.); cyuan@uchicago.edu (C.-S.Y.); 2 Department of Anesthesia and Critical Care, University of Chicago, Chicago, IL 60637, USA; 3 Committee on Clinical Pharmacology and Pharmacogenomics, University of Chicago, Chicago, IL 60637, USA

**Keywords:** epigallocatechin gallate, EGCG, gallic acid, tea polyphenols, flavonoids, anticancer, cell cycle, apoptosis, structure-activity relationship, human colorectal cancer

## Abstract

Green tea is a popular drink consumed daily by millions of people around the world. Previous studies have shown that some polyphenol compounds from green tea possess anticancer activities. However, systemic evaluation was limited. In this study, we determined the cancer chemopreventive potentials of 10 representative polyphenols (caffeic acid, CA; gallic acid, GA; catechin, C; epicatechin, EC; gallocatechin, GC; catechin gallate, CG; gallocatechin gallate, GCG; epicatechin gallate, ECG; epigallocatechin, EGC; and epigallocatechin gallate, EGCG), and explored their structure-activity relationship. The effect of the 10 polyphenol compounds on the proliferation of HCT-116 and SW-480 human colorectal cancer cells was evaluated using an MTS assay. Cell cycle distribution and apoptotic effects were analyzed by flow cytometry after staining with propidium iodide (PI)/RNase or annexin V/PI. Among the 10 polyphenols, EGCG showed the most potent antiproliferative effects, and significantly induced cell cycle arrest in the G1 phase and cell apoptosis. When the relationship between chemical structure and anticancer activity was examined, C and EC did not show antiproliferative effects, and GA showed some antiproliferative effects. When C and EC esterified with GA to produce CG and ECG, the antiproliferative effects were increased significantly. A similar relationship was found between EGC and EGCG. The gallic acid group significantly enhanced catechin’s anticancer potential. This property could be utilized in future semi-synthesis of flavonoid derivatives to develop novel anticancer agents.

## 1. Introduction

Cancer is a major public health problem in the world. One in four deaths in the United States is due to cancer [1]. The clinical management of cancer invariably involves diverse conventional modalities, including surgery, radiation, and chemotherapy [2,3]. Because of the complexity of human cancer, alternative management may be needed to improve the efficacy of therapeutic treatments and the quality of life of patients [4,5]. Cancer chemoprevention or treatment may combine natural products with chemotherapeutic agents to inhibit tumor development [6,7,8].

Botanicals contain bioactive constituents, including some with potential health benefits. Many herbal medicines possess antioxidant properties, which play an important role in therapeutics [9]. Antioxidants are compounds that protect cells against the damaging effects of reactive oxygen species (ROS). When ROS-generating reactions are activated excessively, pathological quantities of ROS are released to create an imbalance between antioxidants and ROS. Oxidative stress has been linked to many different medical conditions, including cancer [10,11]. 

**Figure 1:**
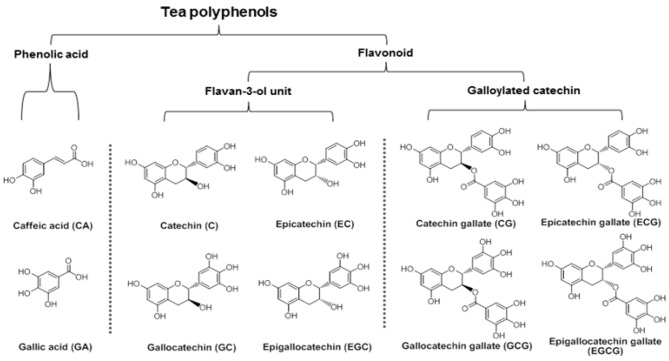
Chemical structures and classifications of tested tea polyphenols.

Green tea, which contains powerful antioxidants, is one of the most popular beverages consumed around the world. Of all the antioxidant compounds found in green tea, the major constituents are polyphenols, including phenolic acids and catechins (Figure 1). Catechins from green tea belong to the family of flavonoids that are powerful antioxidants and free iron scavengers. Many botanical flavonoids possess strong antioxidant activities in the cardiovascular system [12]. Effects of green tea on cancer chemoprevention have been attributed to its antioxidant activities [13,14].

We previously reported that ROS accumulated in ginseng-treated colorectal cancer cells activated a cellular signaling defense pathway, and that ROS levels were reduced by a combination treatment with ginseng and antioxidants [15]. Pilot study data showed that the anticancer activity of panaxadiol (PD), a purified ginseng compound, was enhanced by epicatechin, but not catechin, the two natural antioxidants found in green tea [16]. In a subsequent study, synergistic effects were observed in treating colon cancer cells with a combination of PD and epigallocatechin gallate (EGCG), a major catechin in green tea [17].

EGCG is the most abundant and powerful antioxidant in green tea for cancer chemoprevention [18,19]. Our previous study showed that EGCG enhanced the effects of ginseng compounds in the inhibition of colon cancer cell growth, indicating that green tea could be an effective synergist with anticancer drugs for cancer chemoprevention [17,20]. However, the effects of different tea polyphenols on colon cancer cell growth inhibition have not been systematically compared. In this study, we investigated the antiproliferative effects of 10 major green tea polyphenols using two human colorectal cancer cell lines, HCT-116 and SW-480, and observed that EGCG showed the most potent antiproliferative effects among the tested compounds. The effects of EGCG on the cell cycle and apoptosis were investigated. Structure-activity relationships of tea polyphenols on cancer chemoprevention were also explored.

## 2. Materials and Methods

### 2.1. Chemicals and Materials

Tea polyphenols, epigallocatechin gallate (EGCG), caffeic acid (CA), gallic acid (GA), catechin (C), epicatechin (EC), gallocatechin (GC), epigallocatechin (EGC), catechin gallate (CG), epicatechin gallate (ECG), and gallocatechin gallate (GCG) of biochemical-reagent grade and at least 90% pure, were purchased from Sigma-Aldrich (St. Louis, MO, USA). All cell culture plasticware was purchased from Falcon Labware (Franklin Lakes, NJ, USA) and Techno Plastic Products (Trasadingen, Switzerland). Trypsin, McCoy’s 5A and Leibovitz’s L-15 media, fetal bovine serum (FBS), penicillin/streptomycin solution (200×), phosphate buffered saline, propidium iodide (PI) and RNase were obtained from Mediatech, Inc. (Herndon, VA, USA). A CellTiter 96 Aqueous One Solution cell proliferation assay kit was obtained from Promega (Madison, WI, USA). 

### 2.2. Cell Culture

Human colorectal cancer cell lines HCT-116 (McCoy’s 5A) and SW480 (Leibovitz’s L-15) were obtained from the American Type Culture Collection (Manassas, VA, USA). The cells were grown in the indicated medium supplemented with 5% FBS and 50 IU penicillin/streptomycin in a humidified atmosphere with 5% CO_2_ at 37 °C. When the cells were in the late log/early plateau phase (with about 90% of the surface area covered), healthy and free of contamination, the cell culture medium was removed. After the cells were washed with PBS to remove any trace of serum that would inactivate trypsin, the PBS was discarded. One miniliter of trypsin was added to a 25 mL flask to break the cell-cell and cell-substrate links. Fresh culture medium containing serum (5 mL) was then added to inactivate the trypsin in the cell suspension. After pipetting this suspension, a single-cell suspension was prepared. The cell suspension was then counted for accurate cell density. An aliquot of the cell suspension (1/4 for SW-480, 1/6 for HCT-116) was placed into a new 25 mL flask with the full amount of cell culture medium (10 mL) required for the flask size. The medium was then changed as necessary until the next subculture.

### 2.3. Cell Proliferation Analysis

Tea polyphenols were dissolved in DMSO and stored at −20 °C before use. Cells were seeded in 96-well plates (1 × 10^4^ cells/well). After 24 h, indicated concentrations of drugs were added to the wells. The final concentration of DMSO was 1%. Controls were exposed to culture medium containing 1% DMSO without drugs. Following the indicated incubation period, cell proliferation was evaluated using an MTS assay according to the manufacturer’s instructions. Briefly, the medium was replaced with 100 μL of fresh medium and 20 μL of MTS reagent (CellTiter 96 Aqueous Solution) in each well, and the plate was returned to the incubator for 1–2 h. A 60 μL aliquot of medium from each well was transferred to an ELISA 96-well plate and its absorbance at 490 nm was recorded. Since 1% DMSO did not influence the proliferation of the two cell lines, results were expressed as percent of control (DMSO vehicle set at 100%).

### 2.4. Cell Cycle Analysis

HCT-116 cells were plated at a density of 2 × 10^5^ cells onto 24-well tissue culture plates. After culturing for 1 day, the medium was changed and the tea polyphenols were added. Cells were incubated for 48 h before they were harvested. These cells were fixed gently with 80% ethanol in a freezer for 2 h and were then treated with 0.25% Triton X-100 for 5 min in an ice bath. Cells were resuspended in 150 µL of PBS containing 40 µg/mL propidium iodide (PI) and 0.1 mg/mL RNase. The cells were incubated in a dark room for 20 min at room temperature, and cell cycle analysis was performed using a FACScan flow cytometer (Becton Dickinson, Mountain View, CA, USA) and FlowJo 9.0 software (Tree Star, Ashland, OR, USA). For each measurement, at least 10,000 cells were counted.

### 2.5. Apoptosis Analysis

HCT-116 cells were seeded in 24-well tissue culture plates (2 × 10^5^ cells/well). On the second day, the medium was changed and cells were treated with test compounds. After treatment for 48 h, the cells floating in the medium were collected. The adherent cells were detached with 0.05% trypsin. Then the culture medium containing FBS and floating cells was added to inactivate the trypsin. After being pipetted gently, the cells were centrifuged for 5 min at 1500× *g*. The supernatant was removed and the cells were stained with annexin V-FITC and PI according to the manufacturer’s instructions. Annexin V-FITC detects translocation of phosphatidylinositol from the inner to the outer cell membrane during early apoptosis, and PI can enter the cell in late apoptosis or necrosis. Untreated cells were used as control for the double staining. The cells were analyzed immediately after staining using a FACScan flow cytometer and FlowJo 9.0 software. For each measurement, at least 20,000 cells were counted.

### 2.6. Molecular Dynamics Assay

Using molecular mechanics (MM2) force field method, the molecular dynamics of four galloylated catechins were calculated and their optimized 3D structures were generated. The MM2 force field has been developed for a broad range of chemicals. It is designed to reproduce the equilibrium covalent geometry of molecules [21]. ChemBio 3D Ultra software was used to calculate the total energy of galloylated catechins in the initial state, and each iteration minimizes the total energy by moving the atoms of the system to a position that is energetically more favorable than the previous one. The modeling stops when the system reaches equilibrium.

### 2.7. Statistical Analysis

Data are presented as mean ± standard error (S.E.). A one-way ANOVA was employed to determine statistical significance of results. In some cases, Student’s *t*-test was used for comparing two groups. The level of statistical significance was set at *p* < 0.05.

## 3. Results

### 3.1. Antiproliferative Effects of Tea Polyphenols on Human Colorectal Cancer Cells

**Figure 2:**
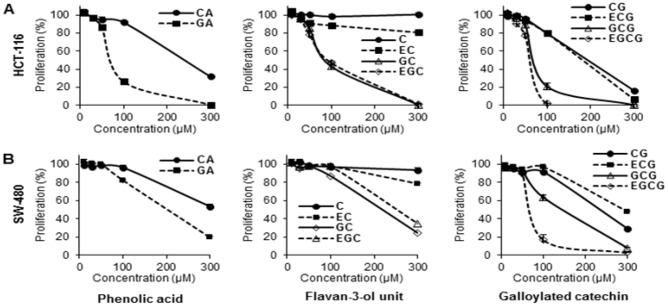
Effects of tea polyphenols on proliferation of human colorectal cancer cells. Cell lines (**A**) HCT-116, and (**B**) SW-480 were employed to evaluate the antiproliferative effects of selected compounds. Cells were treated with 10–300 μM of tea polyphenol compounds for 48 h, and cell proliferation was assayed by MTS method. Results were normalized to each control in percentage and expressed as average ± S.E. of triplicate experiments (solvent vehicle set at 100%).

The main active compounds in green tea are polyphenols. In this study, 10 representative tea polyphenols were selected to evaluate their biological activity. Based on their chemical structures, they were separated into two groups, phenolic acids and flavonoids, the latter of which was separated into two subgroups, the flavan-3-ol unit group and the galloylated catechin group (Figure 1). The antiproliferative effects of the 10 polyphenols were determined, and data are presented based on their structural groups (Figure 2).

As shown in Figure 2A, treatment with 100 µM of the two phenolic acids, CA and GA, HCT-116 cell growth was inhibited by 8.2% ± 2.4% and 73.9% ± 2.9%, respectively. When treatment concentration was increased to 300 µM, more cell growth inhibition was observed in both of the two compounds. For the flavan-3-ol unit group, 100 µM of C, EC, GC and EGC inhibited HCT-116 cell growth by 1.7% ± 1.1%, 11.6% ± 3.2%, 57.0% ± 3.9% and 53.2% ± 2.0%, respectively. The antiproliferative activities of GC and EGC were enhanced by increasing the treatment concentration to 300 µM, but dose-dependent effects were not observed in C and EC. For the galloylated catechin group, 100 µM of CG, ECG, GCG and EGCG inhibited HCT-116 cell growth by 20.2% ± 1.4%, 20.3% ± 1.2%, 79.2% ± 3.4% and 98.4% ± 0.7%, respectively. Dose-dependent effects were observed in all four compounds in this group. Compared to the other 9 tested tea polyphenols, EGCG showed the most potent antiproliferative effects on HCT-116 cells.

The antiproliferative effects of the compounds in SW-480 cells are similar to HCT-116 cells. As shown in Figure 2B, dose-dependent cancer cell growth inhibition was observed on 8 compounds, except C and EC. The effects of many tested tea polyphenols showed lower antiproliferative effects than on HCT-116 cells. Nevertheless, among the 10 test compounds, EGCG again showed the strongest cell inhibition effects on SW-480 cells.

### 3.2. Effects of EGCG on HCT-116 Cell Cycle

**Figure 3:**
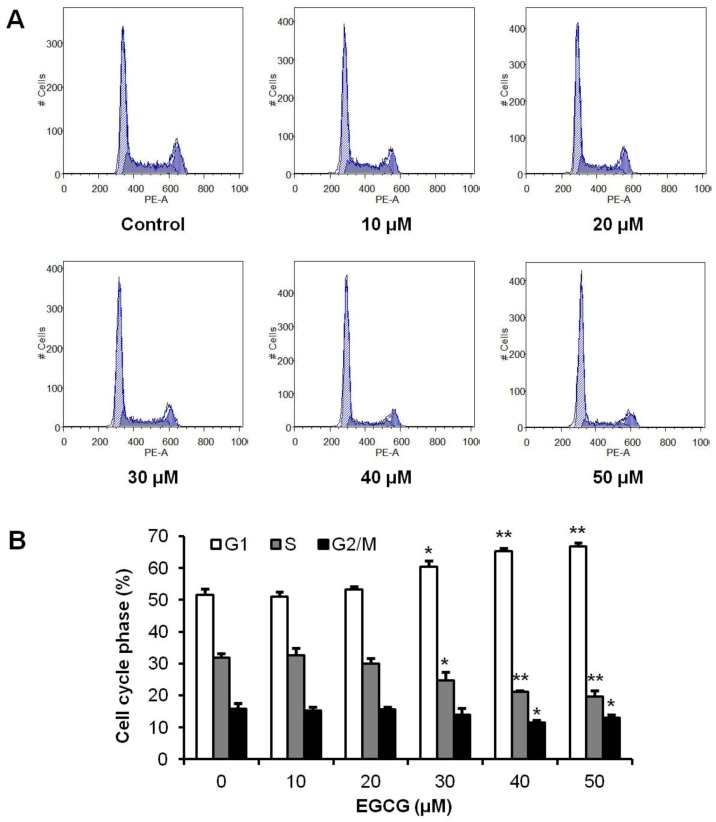
Effects of EGCG on HCT-116 cell cycle. HCT-116 cells were treated with 10–50 μM of EGCG for 48 h, and then cell cycle profile was determined using flow cytometry after staining with PI/RNase. (**A**) Representative histograms of DNA content in each experimental group. (**B**) Percentage of each cell cycle phase with various treatments or with control. Data are presented as the mean ± S.E. of triplicate experiments. * *p* < 0.05; ** *p* < 0.01 *vs.* control.

To evaluate the possible mechanisms by which tea polyphenols inhibited cell growth, the cell cycle profile was assayed by flow cytometry. As shown in Figure 3, the cell cycle profile in the control group was G1 51.6%, S 31.9% and G2/M 15.8%. Treatment with 10–20 µM of EGCG did not change cell cycle profile. After treatment with 30 µM of EGCG, the cell cycle profile was G1 60.3%, S 24.7% and G2/M 14.0%. When treatment concentration increased to 40 µM, cell cycle profile was changed to G1 65.3%, S 21.2% and G2/M 11.6%. Treatment of HCT-116 cells with 50 µM EGCG for 48 h increased G1 phase to 66.8%, compared to 51.6% in vehicle treated cells (*p* < 0.01) (Figure 3B). Thus, EGCG significantly increased the number of cancer cells in the G1 phase.

### 3.3. Apoptotic Effects of EGCG on HCT-116 Cells

**Figure 4:**
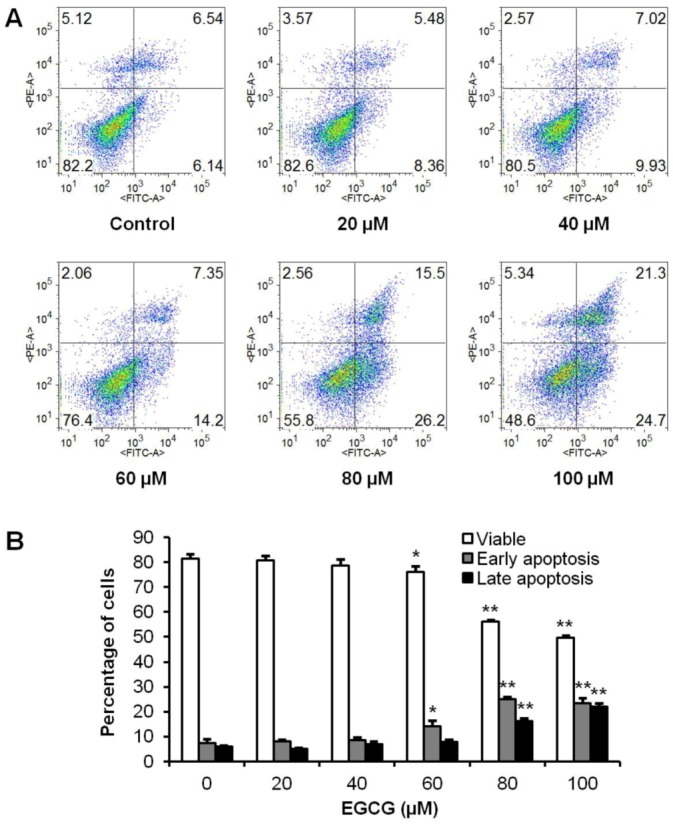
Effects of EGCG on HCT-116 cell apoptosis. HCT-116 cells were treated with 20–100 µM of EGCG for 48 h. Apoptosis was quantified using flow cytometry after staining with annexin V/PI. (**A**) Representative scatter plots of PI (*y*-axis) *vs.* annexin V (*x*-axis). (**B**) Percentage of viable, early and late apoptotic cells. Data are presented as the mean ± S.E. of triplicate experiments. * *p* < 0.05; ** *p* < 0.01 *vs.* control.

To further explore the potential mechanisms by which active tea polyphenol-induced cell death; the apoptotic effects of EGCG were evaluated by flow cytometry after staining with annexin V and PI. Annexin V can be detected in both early and late stages of apoptosis; whereas PI stains cells only in late apoptosis or necrosis. Early apoptotic cells were positive for annexin V and negative for PI (lower right quadrant); late apoptotic or necrotic cells stained for both annexin V and PI (upper right quadrant). The cytogram in Figure 4A shows that incubation with EGCG at 20–40 µM for 48 h did not alter the number of apoptotic cells; which was essentially the same as in the control group. With EGCG at 60–100 µM; cells in early apoptosis were increased. Compared to control (7.3% ± 1.7%); the percentage of early apoptosis increased to 14.1% ± 2.3% (*p* < 0.05); 25.0% ± 1.0% (*p* < 0.01) and 23.5% ± 2.1% (*p* < 0.01) after treatment with EGCG for 48 h at the concentration of 60; 80 and 100 µM; respectively. The induction of late apoptosis was also increased at 80 and 100 µM (Figure 4B). The results demonstrate that EGCG significantly induces cell apoptosis.

### 3.4. Structure-Activity Relationship of Tea Polyphenols in Cancer Chemoprevention

It is interesting to assay the relationship of structures and their biological activities of botanical constituents. In the flavan-3-ol unit group, C and EC showed low or no antiproliferative effects. After an additional phenolic hydroxyl group was attached to C and EC to compose GC and EGC, the antiproliferative effect was increased. The two phenolic acids showed some positive effect, while gallic acid (GA) was more potent than CA. When GA esterified with C and EC to produce CG and ECG, their antiproliferative effect was increased significantly. A similar relationship was also observed in the GC and EGC. GC and EGC showed antiproliferative effects in a dose-dependent manner. After GA esterified with them to compose GCG and EGCG, their antiproliferative effects were further increased. Overall, except for C and EC, the tea polyphenols possess different cancer cell inhibition potentials, and EGCG showed the most potent antiproliferative activities. On the other hand, GA showed some antiproliferative effects. When other catechins were esterified with GA, their antiproliferative potential was increased significantly. This could be a regular pattern in the relationship of structural-anticancer effects of tea catechins.

### 3.5. Molecular Modeling of EGCG and Other Galloylated Catechins

**Figure 5:**
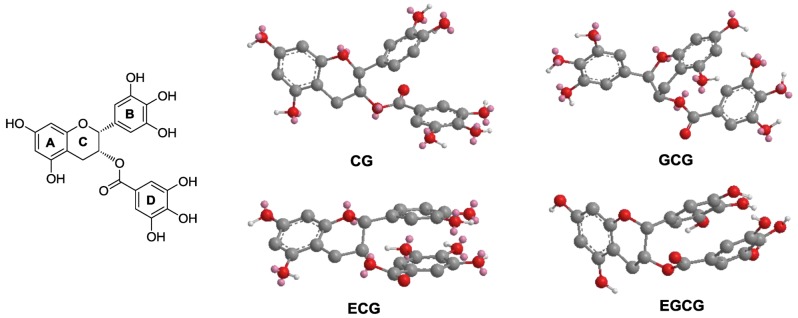
The three-dimensional (3D) structures of galloylated catechins. Molecular modeling of EGCG and other galloylated catechins was generated by MM2 force field method. (Left panel) Taking EGCG as a model structure, the three aromatic rings are assigned as rings A, B and D, while the pyran ring is assigned C. (Right panel) MM2 optimized 3D structures of CG, GCG, ECG and EGCG are shown.

As shown in Figure 5, the optimized 3D structures of EGCG and other galloylated catechins were generated by the MM2 force field method [21]. The stereo configuration of EGCG (2*R*,3*R*) and GCG (2*R*,3*S*) are different but the calculated 3D structures of them are quite similar. As shown in Figure 5 (Left), we assigned each ring with A, B, C and D in the galloylated catechins group. Relative to the spatial arrangement of the three aromatic rings, the pyran ring C centrally links the rings. Two aromatic rings are parallel and relatively close and could be formed as an intermolecular interaction, leaving the third aromatic ring vertical to the two parallel rings. Ring C links three aromatic rings with a position deep inside, making the whole molecule concave in the center, keeping the three aromatic rings close to each other. As we assayed above, phenolic hydroxyl groups on rings B and D are positive for anticancer activity. These structure-activity relationships and molecular modeling observations could be utilized in the future semi-synthesis of tea catechin derivatives to develop more potent anticancer agents in this series of compounds.

## 4. Discussion

Tea from *Camellia sinensis* L. of the Theaceae family is one of the most ancient and widely consumed beverages in the world. Tea can be classified into three types: green, oolong, and black. Green tea, which is produced from non-fermented leaves and derived directly from drying fresh tealeaves, is a popular drink consumed every day by hundreds of millions of people. In Oriental cultures, it has been widely believed for a long time that green tea has medicinal efficacy for the prevention and treatment of many diseases. Modern scientific studies of biological and pharmacological properties, however, were started only recently [22,23], and much attention has been focused on its antioxidant potential, including cardiovascular protection, antimutagenic, antiviral and anticancer activities [16,24]. 

It is believed that the main bioactive constituents of green tea are polyphenols, including phenolic acids and flavonoids (Figure 1). Flavan-3-ols or catechins are the most predominant compounds in green tea accounting for 16%–30% of the dry weight. EGCG is the major catechin in tea. On the basis of recent studies, previous data suggested that EGCG is responsible for much of the biological activity mediated by green tea, including cancer chemoprevention. Tea polyphenols may have adjuvant potential to increase other anticancer compounds’ effects. Panaxadiol, a pseudoaglycone of diol-type triterpenoid with a dammarane skeleton, is an active anticancer compound in steamed ginseng [7]. In our previous study, a beneficial effect was obtained when combining panaxadiol with EC in the treatment of colorectal cancer cells [16]. Recently, we observed that EGCG significantly enhanced antiproliferative effect of panaxadiol on human colorectal cancer cells [17].

Anticancer activities of some tea polyphenols were evaluated [25]. Tea polyphenols possess chemopreventive effects on prostate [26], breast [27], lung [28] and colorectal cancer [29]. However, systemic structure-activity relationship study was limited. In this study, we selected 10 tea polyphenols, which were representative compounds in three groups/subgroups, and explored the relationships of their structures with anticancer potentials.

Using two colorectal cancer cell lines HCT-116 and SW-480, the antiproliferative effects of the tea polyphenols were evaluated. The two cell lines used in this study have varied p53 expression: HCT-116 is p53 wild type, while SW-480 cells contain a p53 mutation. Cancer cells with p53 mutations are resistant to many chemotherapeutic agents. Overall, the growth inhibition for almost all tested compounds in HCT-116 cells was stronger than in SW-480 cells (Figure 2), suggesting that p53 may play an important role in the inhibitory effect of tea polyphenols on colorectal cancer cell growth. 

In this study, among 10 tested polyphenols, EGCG showed the most potent antiproliferative effects. The antiproliferative effect of EGCG is even more potent than 5-fluorouracil, which is a chemotherapy drug on colorectal cancer [30]. To explore possible anticancer mechanisms of EGCG, we performed cell cycle and apoptotic analysis. Cell cycle assays showed that EGCG could increase cell percentage in the G1 phase, which may in part contribute to the cancer cell growth inhibition of EGCG (Figure 3). Apoptosis is considered an important pathway in the inhibition of tumor growth by many anticancer agents [31]. We observed that EGCG induced significant cell apoptosis in HCT-116 cells (Figure 4), suggesting that the antiproliferative effects of EGCG in human colorectal cancer cells is at least in part through the apoptosis pathway. 

Based on the chemical structures and observed biological activities, we explored structure-activity relationship analysis of tea polyphenols on cancer chemoprevention. Within the three groups/subgroups, phenolic acids showed relatively lower antiproliferative effects compared to active flavonoids, while the effects of gallic acid were more potent than those of caffeic acid. For the 8 flavonoids, in each subgroup, galloylated catechins showed even more potent activity. For example, the antiproliferative effects of GC, EGC, GCG and EGCG were stronger than their corresponding compounds C, EC, CG and ECG, respectively. Interestingly, by comparing antiproliferative effects with their structures between the two subgroups of flavonoids, we found that when a catechin unit is esterified with gallic acid, its antiproliferative effect is increased significantly. In addition, though the stereo configuration of tea polyphenols is different, their optimized 3D pharmacophore structures are quite similar. The two close parallel aromatic rings and a third aromatic ring vertical to the two parallel rings may play a key role in their biological activities. This molecular dynamic information of tea catechins could help future structural modifications on this group of compounds.

## 5. Conclusions

In this study, we evaluated the chemopreventive effects of 10 tea polyphenols on human colorectal cancer cells. Many tested tea polyphenols exert dose-dependent antiproliferative effects, and EGCG showed the most potent activities. Cell cycle arrest and apoptosis induction may play an important role in EGCG-induced cancer cell death. Structure-activity relationship analysis showed that if gallic acid is esterified with catechins, their anticancer activities increased significantly. Molecular dynamics assay on EGCG suggested that two close parallel aromatic rings and a third aromatic ring vertical to the two parallel rings may play a key role in the pharmacophore activity. Data from this study supplied new clues for future semi-synthesis of tea catechin derivatives to develop novel anticancer agents.
